# Patellar Tendon Reconstruction Following Septic Arthritis: A Case Report of an Autograft Technique With Quadriceps Tendon Reinforcement

**DOI:** 10.7759/cureus.109307

**Published:** 2026-05-20

**Authors:** Josue M Portillo Valle

**Affiliations:** 1 Orthopaedics, Hospital Clínic de Barcelona, Barcelona, ESP

**Keywords:** extensor mechanism reconstruction, hamstring autograft, patellar tendon defect, septic arthritis, v-y quadriceps plasty

## Abstract

Loss of the patellar tendon or the entire extensor mechanism is a rare but devastating complication of post-traumatic septic arthritis. Although both autografts and allografts are used for reconstruction, the combination of hamstring tendon autografts with quadriceps tendon reinforcement offers a reliable biological option to restore knee extension.

We report the case of a 35-year-old man who developed complete patellar tendon loss following septic arthritis secondary to a heavily contaminated penetrating knee injury from a motorcycle accident. After infection control with aggressive debridement, partial patellectomy, and 45 days of intravenous antibiotics, reconstruction was performed using semitendinosus and gracilis autografts passed through transosseous tunnels in the tibial tuberosity and patella, reinforced with a V-Y quadriceps tendon plasty.

At the one-year follow-up, the patient achieved good functional outcomes: Lysholm score of 88, International Knee Documentation Committee (IKDC) score of 82, Kujala score of 86, and Tegner score of 5. He returned to heavy manual labor without limitations, despite not resuming competitive sports. This case demonstrates that biological reconstruction with hamstring autografts and quadriceps reinforcement can successfully restore extensor mechanism function after post-traumatic septic arthritis. In previously infected fields, autografts offer improved biological incorporation and a lower theoretical risk of reinfection, while providing a cost-effective alternative to allograft use.

## Introduction

Septic arthritis most commonly affects the knee. This destructive inflammatory process results from direct bacterial invasion of the joint space and carries an incidence of 2 to 6 cases per 100,000 population annually. In certain regions, the associated mortality approaches 7.05% [1-5]. Infection may follow penetrating trauma, open wounds, prior joint surgery, or hematogenous seeding from a distant focus.

Major complications include irreversible cartilage damage, osteomyelitis, joint stiffness, and permanent loss of knee function. Among these, disruption of the extensor mechanism has been reported in 25% to 50% of severe or destructive cases of septic arthritis in patients older than 50 years undergoing surgical debridement [6]. Aggressive debridement, although necessary for infection control, may require sacrifice of critical structures needed for normal knee extension [7]. In such situations, reconstruction with either autograft or allograft tissue becomes essential to restore extensor continuity. Although allografts can be employed as an alternative when autologous tissue is unavailable, their use continues to raise concerns regarding complications. Balato et al. documented considerable rates of graft failure and postoperative infection in patients who underwent extensor mechanism reconstruction using allograft tissue following total knee arthroplasty. These observations underscore the importance of judicious graft selection and support consideration of autograft techniques in younger or higher-demand patients whenever feasible [8-10].

This case report presents the use of hamstring tendon autografts combined with quadriceps tendon V-Y plasty to reconstruct the extensor mechanism in a patient with patellar tendon loss following post-traumatic septic arthritis, together with the clinical outcome at one year.

## Case presentation

A 35-year-old man presented to the ED six hours after a motorcycle accident. He had received no prehospital care and lived in a rural community located four hours from the nearest hospital.

**Figure 1:**
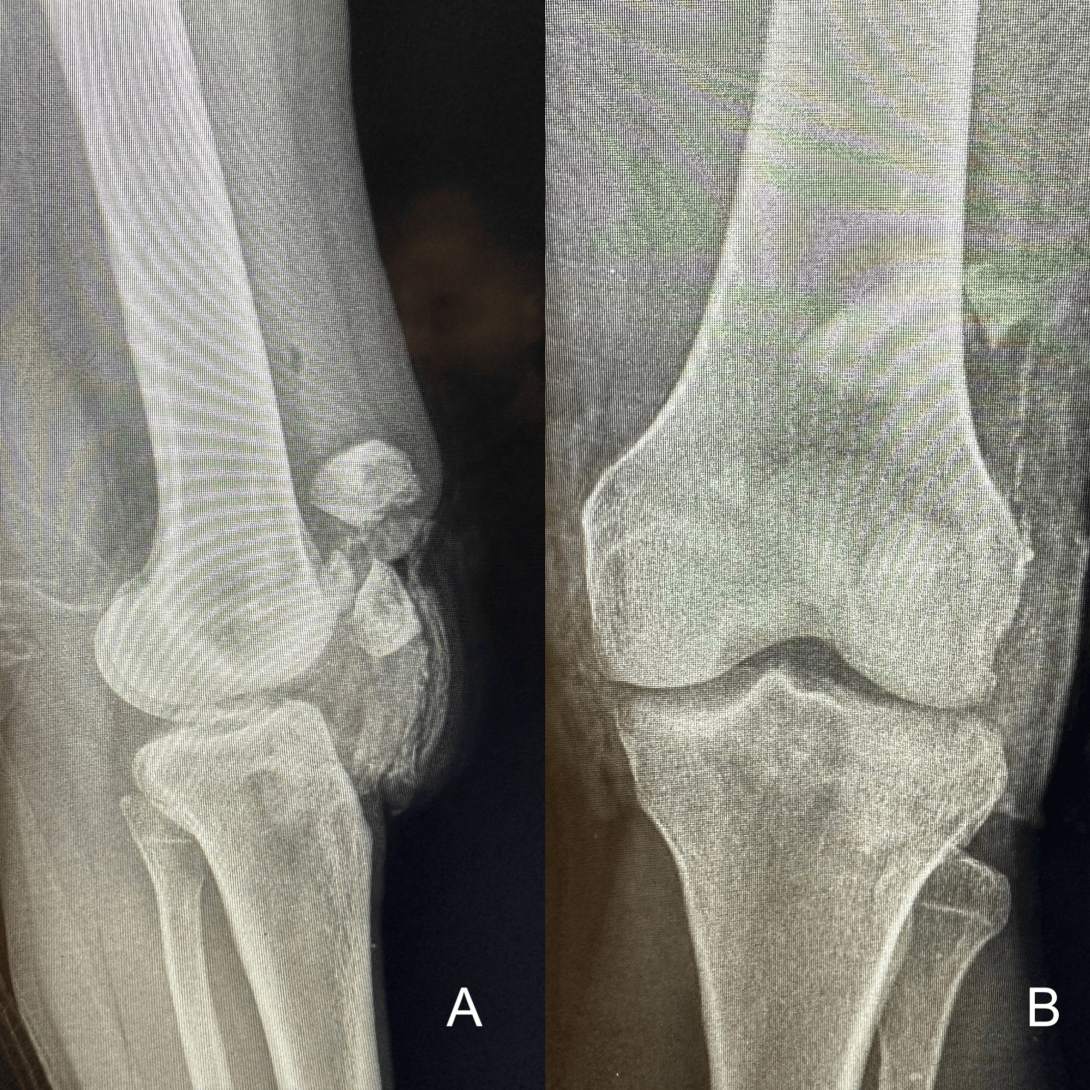
Radiographs obtained on admission to the ED. (A) Anteroposterior view showing a transverse patellar fracture. (B) Lateral view of the knee.

Physical examination revealed a 5-cm penetrating wound over the knee containing grass, mud, and other debris. Triple IV antibiotic prophylaxis with cefazolin, gentamicin, and penicillin was administered, and the wound was thoroughly irrigated with 20,000 mL of normal saline, with removal of all visible foreign material. Plain radiographs demonstrated a transverse patellar fracture classified as AO/OTA 34-C1. The injury was classified as a Gustilo-Anderson type IIIA open fracture due to the high-energy mechanism, 5-cm penetrating wound with heavy contamination, including grass, mud, and debris, and delayed presentation (Figure 1).

**Figure 2:**
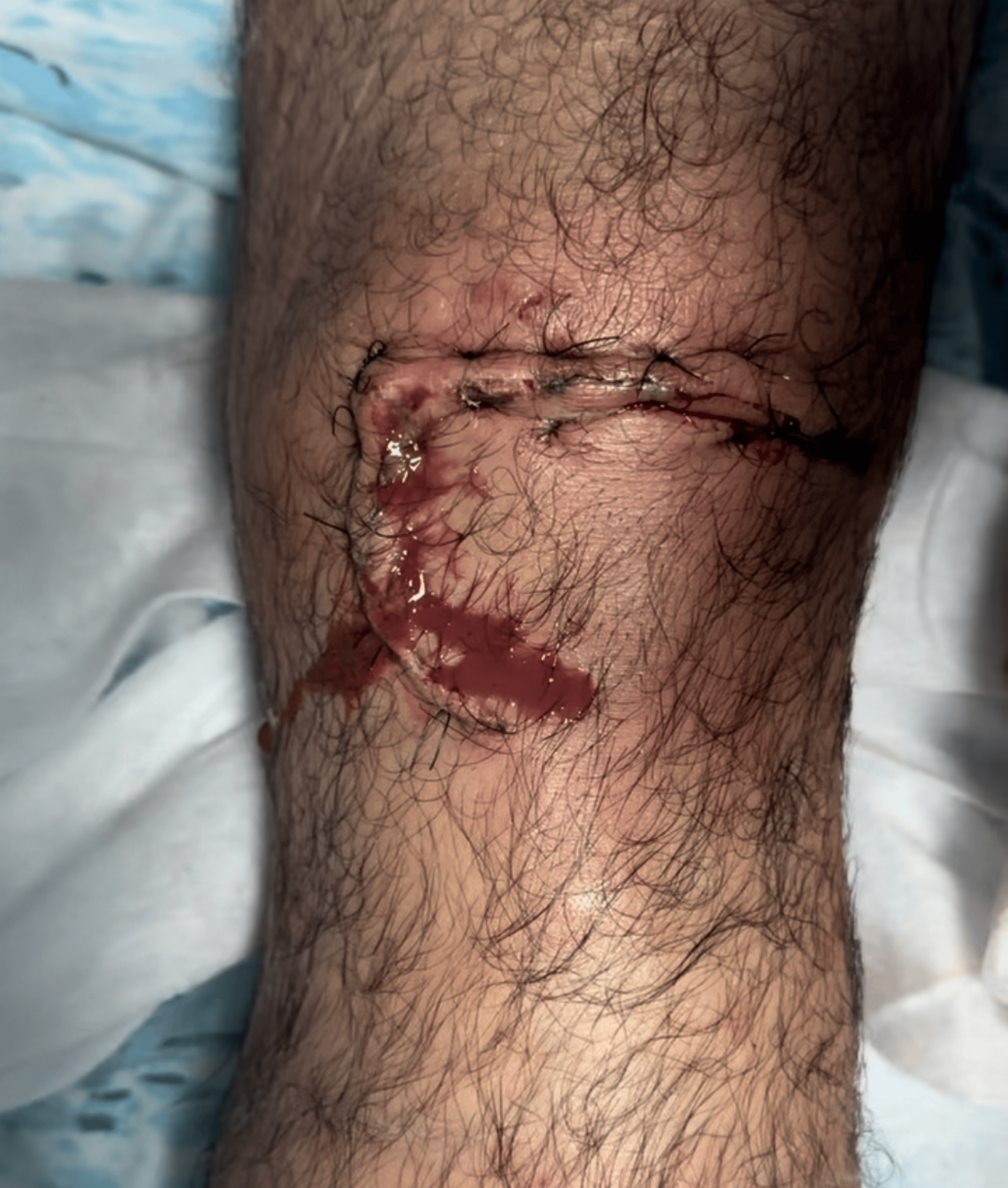
Wound appearance 48 hours after the first surgery. Clinical photograph showing the wound 48 hours after the initial irrigation and debridement.

**Figure 3:**
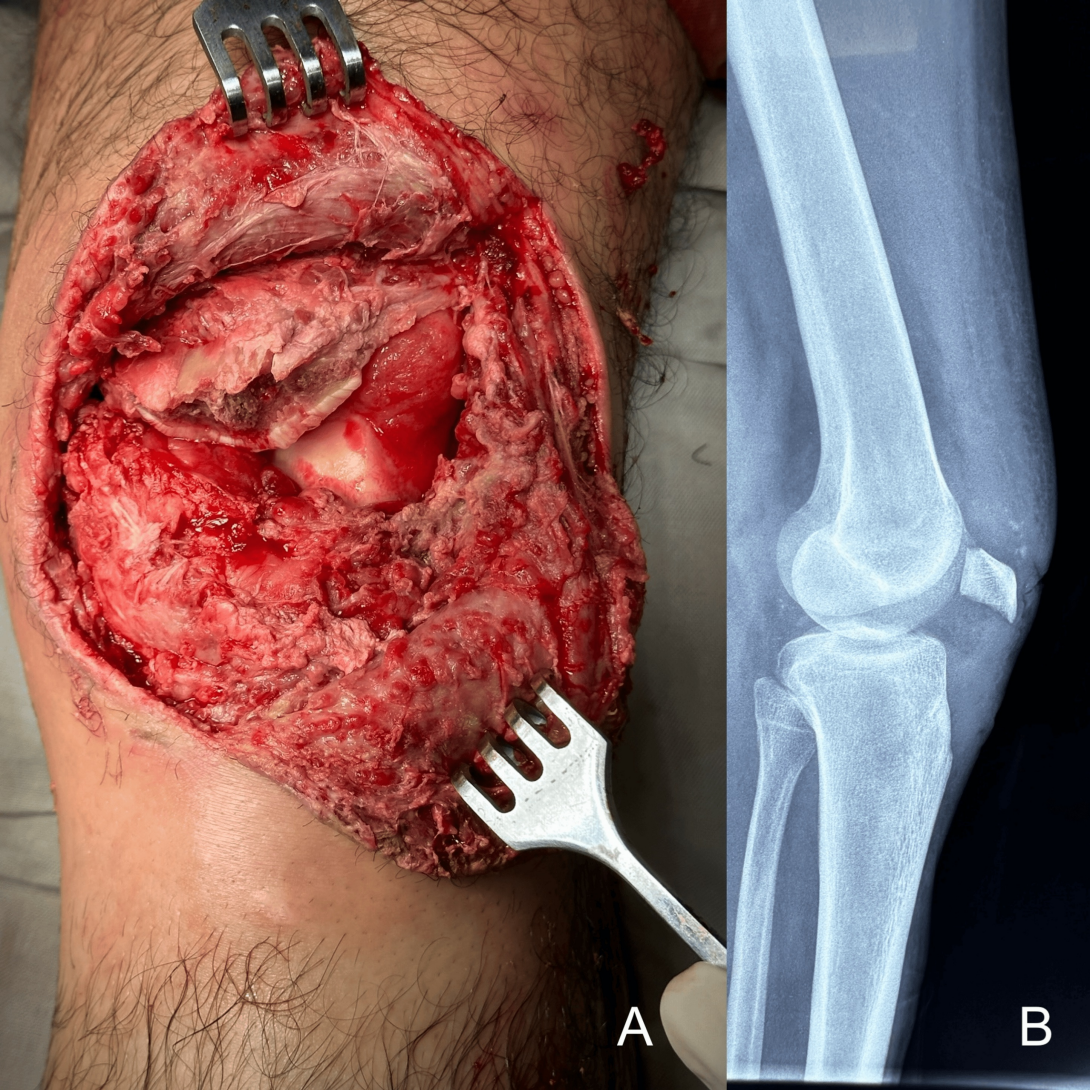
Intraoperative photographs and radiographs obtained during the second surgical debridement. (A) Intraoperative view after aggressive debridement and excision of infected soft tissue. (B) Lateral radiograph showing the residual patellar fragments following extensive debridement.

After hemodynamic stabilization and laboratory evaluation, the patient underwent urgent surgical irrigation, debridement, and management of the traumatic arthrotomy. Forty-eight hours later, the patient developed persistent wound drainage. On clinical examination, the knee was markedly swollen, with significant periwound erythema, seropurulent discharge, and visible areas of necrotic soft tissue at the wound edges (Figure 2), necessitating a return to the operating room for further debridement. Intraoperatively, discoloration of the patellar tendon and adjacent patella was evident, requiring partial patellectomy and resection of the non-viable tendon to achieve adequate source control (Figure 3).

The patient then received 45 days of broad-spectrum IV antibiotics. Intraoperative cultures obtained during the initial irrigation and debridement, the second debridement, and immediately before reconstruction were all negative, with no microorganism isolated. Progressive clinical improvement was noted, culminating in complete resolution of the infection. Once the joint appeared clinically sterile, reconstruction of the patellar tendon was performed as detailed below.

Surgical technique

Patient Positioning and Preparation

Under general anesthesia, the patient was positioned supine on a radiolucent table with lateral supports and foot holders to permit controlled knee flexion at 30°, 60°, and 90°. Standard skin preparation and draping were performed, and a thigh tourniquet was inflated as proximally as possible.

Approach and Identification of the Injury

A longitudinal midline incision was made from 1 cm distal to the tibial tuberosity to the inferior pole of the patella. Dissection proceeded through the tissue planes to fully expose the patellar tendon defect. All fibrotic remnants and peritendinous scar tissue were carefully excised.

Identification and Harvesting of Autografts

With the knee flexed at 60°, the previous incision was extended approximately 4 cm distally to access the pes anserinus. After entering the bursa and identifying the medial collateral ligament insertion, the semitendinosus and gracilis tendons were harvested while preserving their distal attachments. Residual muscle tissue was removed using scissors or a periosteal elevator.

Reconstruction of the Extensor Mechanism

For reconstruction, the medial and lateral borders of the tibial tuberosity were exposed. A transverse tibial tunnel was created approximately 1 cm posterior to the tuberosity prominence under direct visualization. The bundle diameter was measured to determine the appropriate tunnel size. A guidewire was placed from lateral to medial and overdrilled with a cannulated reamer according to bundle thickness. Each bundle was then passed through the transverse tunnel to the contralateral side so that the bundle descending on one side returned proximally on the opposite side. The semitendinosus tendon was secured at both the medial entrance and lateral exit using ultra-high-molecular-weight polyethylene suture. The grafts were then routed toward the patella. The semitendinosus was passed proximally through a lateral channel and the gracilis through a medial channel. Both tendons were subsequently passed through a patellar tunnel in opposite directions using a passing suture. Patellar height and graft tension were assessed intraoperatively by adjusting tension with the knee flexed at 30° and confirming central patellar tracking in the trochlear groove, together with normal patellar position, with the inferior pole aligned appropriately relative to the tibial plateau to avoid patella baja or alta.

**Figure 4:**
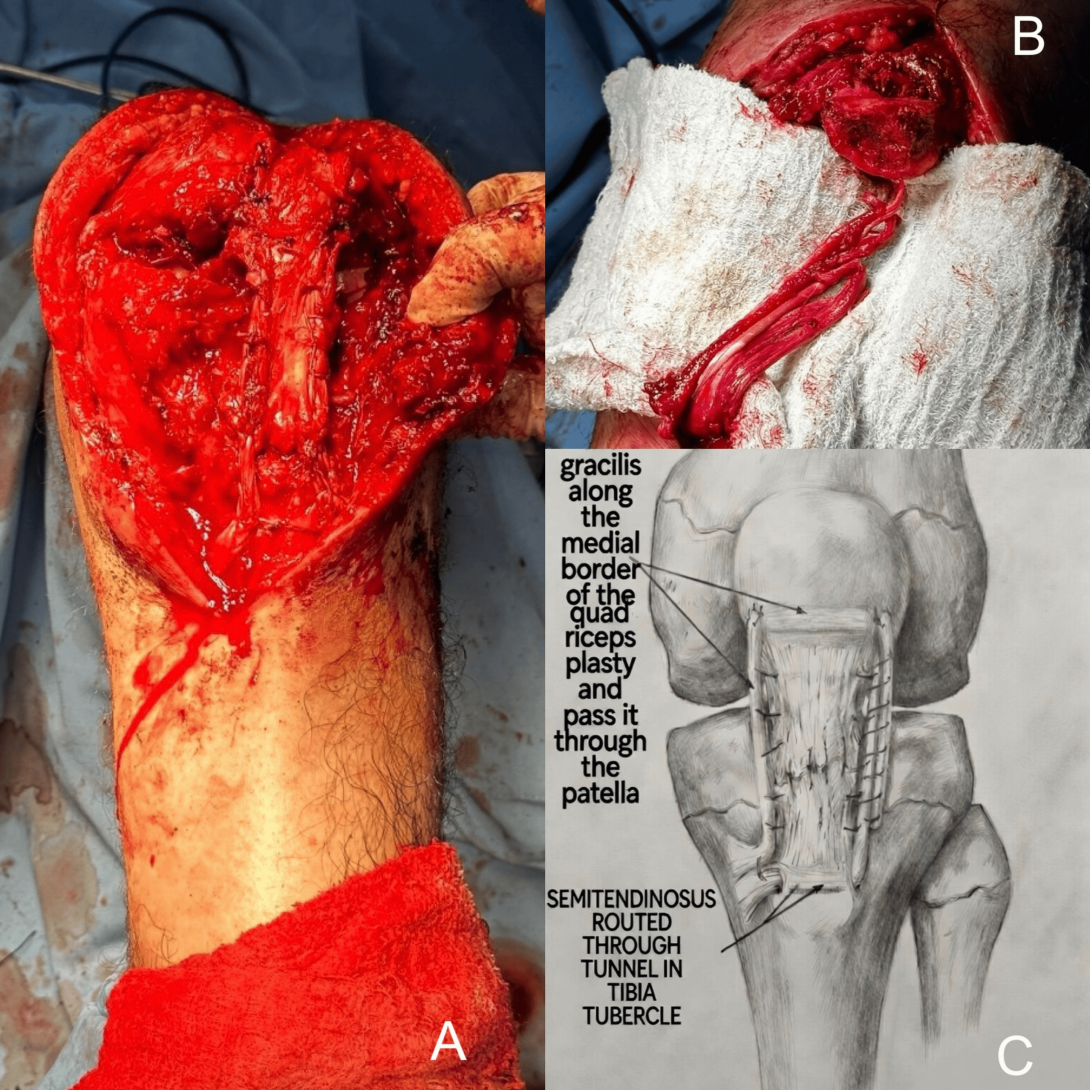
Intraoperative images of extensor mechanism reconstruction. (A) V-Y quadricepsplasty reinforced with semitendinosus and gracilis tendon autografts. (B) Harvesting of the semitendinosus and gracilis tendons with preservation of their distal tibial insertion. (C) Schematic representation of the surgical technique for patellar tendon reconstruction using hamstring autografts passed through transosseous tunnels and reinforced with V-Y quadricepsplasty.

A V-Y plasty of the quadriceps tendon was then performed to reinforce the reconstructed patellar tendon with native quadriceps tissue. Final fixation was achieved with Krackow sutures using non-absorbable No. 2 Ultrabraid (Smith & Nephew). Full passive knee range of motion was confirmed without evidence of graft over-tensioning or overload. The tourniquet was released, deep tissues were closed in layers with Vicryl 0 and 2-0 sutures, and the skin was approximated with a subcuticular Monocryl suture. A sterile dressing was applied, and the knee was immobilized in full extension with a brace (Figure 4) [11-14].

Postoperative management

Immediate weight-bearing as tolerated was allowed following reconstruction, with the knee locked in full extension using a hinged knee brace. Early weight-bearing is important in extensor mechanism reconstruction to prevent joint stiffness, minimize quadriceps muscle atrophy, and promote synovial fluid circulation for cartilage nutrition, thereby optimizing long-term functional recovery. To protect the repair in this post-infectious field, the knee remained locked in full extension for the first two weeks. Progressive flexion was then introduced up to 45° for an additional two weeks.

**Figure 5:**
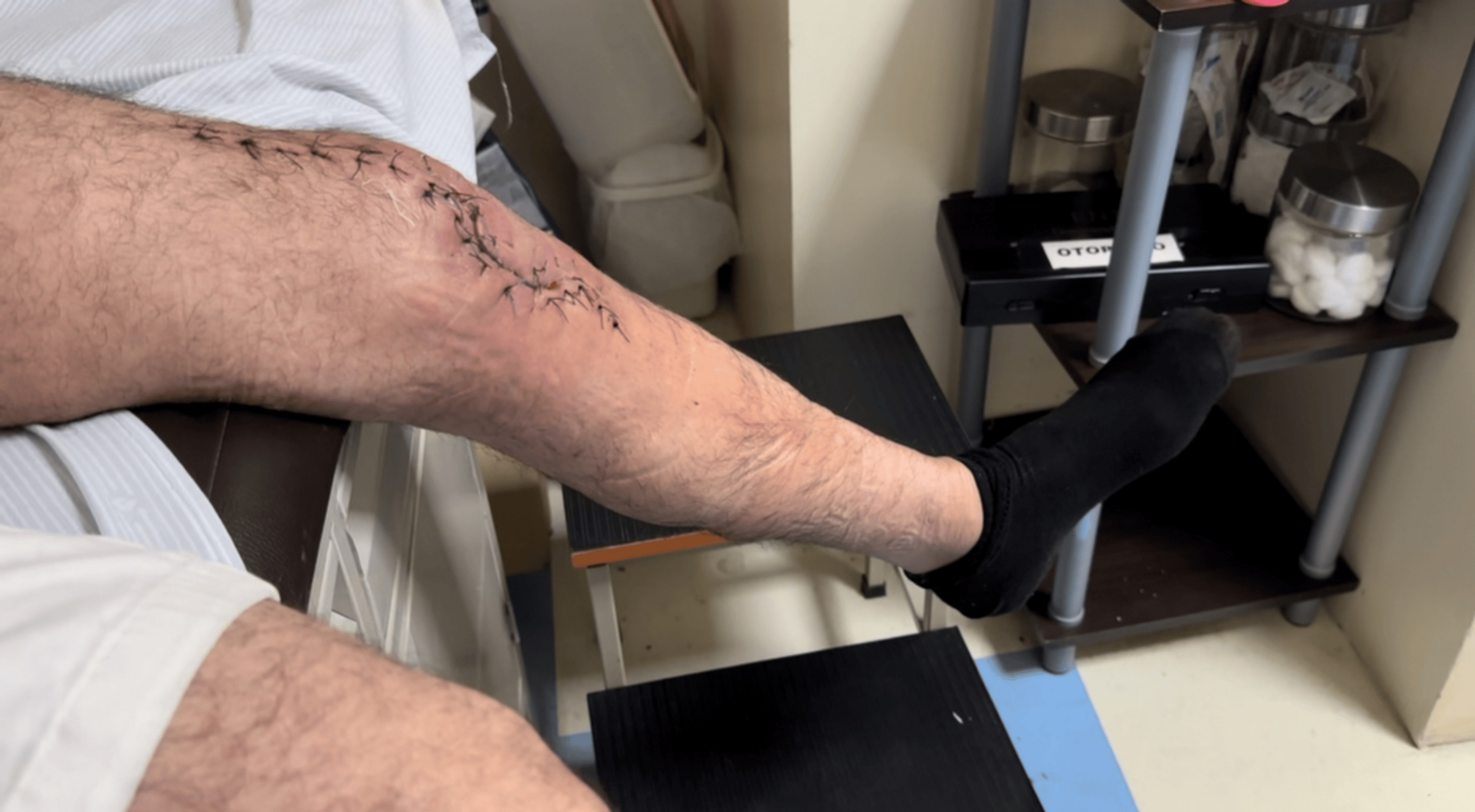
Clinical appearance two weeks postoperatively. Clinical photograph obtained two weeks after surgery, during suture removal. The patient demonstrates active knee extension and performs isometric quadriceps strengthening exercises.

**Figure 6:**
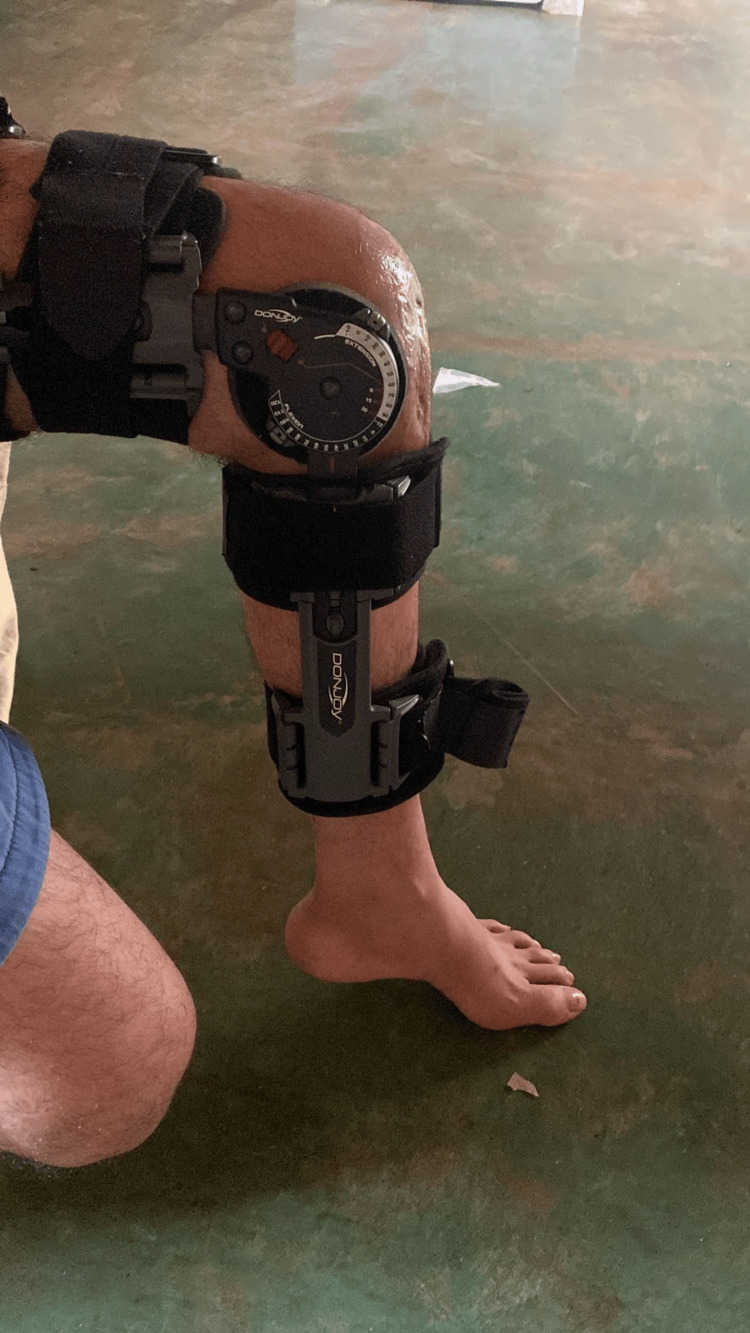
Clinical appearance six weeks postoperatively. Clinical photograph obtained six weeks postoperatively, demonstrating 90° of knee flexion.

At one month postoperatively, flexion was advanced to 90°, and active-assisted range-of-motion exercises were initiated. Early postoperative clinical recovery is shown in Figures 5-6. Although a theoretical risk of periostitis or stress reaction around the transosseous tunnels exists in a post-infectious field with potentially compromised bone quality, serial clinical and radiographic evaluations at six weeks and three months showed no signs of periostitis, tunnel widening, bone resorption, or stress reaction.

At the one-year follow-up, the patient demonstrated excellent clinical progress, with a mild residual extension lag of 10° and knee flexion of 100°. The Lysholm Knee Scoring Scale score was 88 points, the International Knee Documentation Committee (IKDC) Subjective Knee Form score was 82 points, and the Kujala Anterior Knee Pain Scale score was 86 points. Although he did not return to competitive sports, he was able to resume heavy manual labor without restrictions. These results support successful restoration of extensor mechanism function and adequate knee performance for demanding daily activities [15-19].

## Discussion

The present case demonstrates that complete patellar tendon loss resulting from post-traumatic septic arthritis can be successfully managed through a staged biological reconstruction strategy combining hamstring tendon autografts with V-Y quadriceps plasty reinforcement. The key determinants of success in this case were aggressive and repeated surgical debridement, prolonged intravenous antibiotic therapy until clinical and microbiological eradication was confirmed, and subsequent single-stage biological reconstruction once a sterile field was established. At the one-year follow-up, the patient achieved satisfactory functional scores across multiple validated outcome measures and returned to demanding occupational activities, despite a mild residual extension deficit.

Septic arthritis following heavily contaminated penetrating knee trauma represents one of the most challenging scenarios in orthopaedic surgery. The combination of a high-energy mechanism, delayed presentation, and gross organic contamination, as observed in our patient, creates conditions that strongly predispose to aggressive polymicrobial infection and rapid soft tissue destruction [1-5]. Although all intraoperative cultures obtained in this case remained negative, the clinical presentation was consistent with established criteria for septic arthritis, and the decision to proceed with aggressive serial debridement followed by prolonged antibiotic therapy was appropriate and consistent with current evidence-based guidelines [7,20]. The negative culture results may reflect prior antibiotic administration, limitations of sampling techniques, or the presence of biofilm-producing organisms that are poorly detected by conventional culture methods.

Compared with previously published reports, our findings are broadly consistent with the experience of Andersen RC et al. [13], who analyzed open, combat-related extensor mechanism disruptions and emphasized that thorough serial debridement is the cornerstone of successful infection control before reconstruction. In their series, patients required a mean of 11 surgical procedures, and a substantial proportion ultimately required allograft reconstruction or arthrodesis, outcomes that underscore the severity of this injury pattern and highlight the favorable course achieved in our patient through single-stage autograft reconstruction following only two debridement procedures. This difference may be partly explained by the relatively contained nature of the infection in our case, despite heavy contamination, as well as by the early initiation of triple intravenous antibiotic therapy and copious intraoperative irrigation.

Regarding the reconstruction technique, Mouarbes D et al. [15] recently described the reverse double figure-of-eight technique using hamstring tendons for chronic patellar tendon rupture in native knees, reporting satisfactory functional outcomes and confirming the mechanical reliability of hamstring autografts as a reconstructive substrate for the extensor mechanism. Their results support the rationale for using semitendinosus and gracilis tendons in cases involving significant tissue loss, where the native tendon cannot be primarily repaired. Similarly, Mihalko WM et al. [12] demonstrated in a cadaveric biomechanical study that patellar tendon repair augmented with hamstring autograft provides construct stiffness and failure load comparable to those of the intact native tendon, providing a biomechanical basis for the technique employed in our patient. Fortier LM et al. [9], in a systematic review of patellar tendon restoration techniques, concluded that reconstruction is generally favored over repair in cases involving substantial tissue deficiency, chronic rupture, or compromised tissue quality, all conditions present in our case, and reported acceptable functional outcomes across multiple reconstruction methods. The addition of a V-Y quadriceps plasty as a reinforcement layer, as performed in our patient, has been previously described as a useful adjunct to increase construct strength, reduce tension on the primary reconstruction, and provide an additional vascularized biological layer that may promote graft incorporation, an advantage of particular relevance in a previously infected and potentially hypovascular soft tissue environment [13,14].

From a clinical practice standpoint, this case supports several important principles in the management of complex extensor mechanism defects in post-infectious settings. First, infection eradication must be confirmed before reconstruction and not merely assumed on the basis of clinical improvement alone. In our patient, this required 45 days of broad-spectrum intravenous antibiotics and three sets of negative intraoperative cultures before reconstruction was deemed safe. Serological markers of infection, including C-reactive protein and erythrocyte sedimentation rate, should be monitored serially and normalized before proceeding, a practice that should be standardized in future protocols. Second, the use of transosseous tunnels in both the tibial tuberosity and the patella, combined with preservation of the distal hamstring insertions, provides secure fixation without the need for metallic implants, thereby reducing the risk of implant-related infection recurrence and lowering procedural cost. This is particularly relevant in resource-limited or rural settings where allograft availability may be restricted and implant cost is a barrier to care. Third, early protected weight-bearing with a locked knee brace, as employed in this case, appears to be safe and may contribute to preventing joint stiffness and quadriceps atrophy during the early postoperative period, consistent with the principles outlined by West JL et al. [16] regarding early motion protocols following extensor mechanism repairs. Fourth, multidisciplinary collaboration with infectious disease specialists, who are responsible for guiding antibiotic selection, duration, and de-escalation, was integral to the successful outcome and should be considered an important component of the management pathway in similar cases.

Despite the favorable outcome observed in this case, several limitations merit consideration. From a technical standpoint, the procedure demands meticulous surgical execution, including precise tunnel placement and appropriate graft tensioning, which may limit its reproducibility outside specialized centers. Regarding the graft itself, harvesting the semitendinosus and gracilis tendons introduces donor-site morbidity, and the combined graft diameter may be mechanically insufficient to fully replicate the native patellar tendon in high-demand patients. This may partly explain the residual 10° extension lag observed at the one-year follow-up, and whether this deficit will resolve with continued rehabilitation or persist long term remains to be determined. Beyond the technical aspects of this case, important questions remain unanswered in the literature: the optimal timing of reconstruction following infection eradication, the minimum antibiotic duration required before safe reconstruction, and the long-term durability of this technique in post-infectious settings are all issues that cannot be resolved by a single case report. Larger prospective series with standardized outcome measures are needed to establish evidence-based guidelines for this rare but devastating complication.

## Conclusions

Prompt treatment of penetrating knee injuries is essential. Adequate prehospital care and early aggressive management can help prevent the severe complications illustrated in this case. Timely reconstruction using a biological technique may optimize functional recovery and minimize long-term disability. This case further emphasizes that optimal outcomes following complex extensor mechanism reconstruction in a post-infectious setting rely on a multidisciplinary approach, including close collaboration with infectious disease specialists and a structured rehabilitation program. Nevertheless, as this is a single-case report with only one-year follow-up, the results should be interpreted with caution. Larger series with longer-term follow-up are needed to confirm the generalizability and durability of this technique in post-infectious extensor mechanism defects.
